# Mesenchymal stem cells modified by FGF21 and GLP1 ameliorate lipid metabolism while reducing blood glucose in type 2 diabetic mice

**DOI:** 10.1186/s13287-021-02205-z

**Published:** 2021-02-15

**Authors:** Binghua Xue, Xiuxiao Xiao, Tingting Yu, Xinhua Xiao, Jing Xie, Qiuhe Ji, Li Wang, Tao Na, Shufang Meng, Lingjia Qian, Haifeng Duan

**Affiliations:** 1 Department of Military Cognitive and Stress Medicine, Institute of Military Cognitive and Brain Sciences, Academy of Military Sciences, Beijing, 100850 China; 2 Department of Experimental Hematology, Beijing Institute of Radiation Medicine, Academy of Military Sciences, Beijing, 100850 China; 3 Department of Endocrinology, Chinese Academy of Medical Sciences and Peking Union Medical College, Peking Union Medical College Hospital, Beijing, 100730 China; 4 Department of Endocrinology and Metabolism, Xijing Hospital of Airforce Medical University, Xi’an, 710032 Shanxi China; 5 The Cell Collection and Research Center, Key Laboratory of the Ministry of Health for Research on Quality and Standardization of Biotech Products, National Institutes for Food and Drug Control, Beijing, 100050 China

**Keywords:** Type 2 diabetes mellitus, Mesenchymal stem cell, FGF21, GLP1

## Abstract

**Objective:**

The purpose of this study was to investigate the therapeutic effects of genetically modified mesenchymal stem cells (MSCs) in the treatment of type 2 diabetes mellitus (T2DM) in order to identify a new method for treating diabetes that differs from traditional medicine and to provide a new means by which to fundamentally improve or treat diabetes.

**Methods:**

MSCs derived from adipose tissue were modified to overexpress FGF21 and GLP1, which was achieved through lentiviral particle transduction. The cells were transplanted into BKS.Cg-Dock7m+/+Leprdb/Nju mice (T2DM mouse model). Injections of physiological saline (0.1 mL) and liraglutide (0.5 mg/kg) were used as negative and positive controls, respectively. ELISA or Western blotting was used for protein analysis, and quantitative real-time PCR was used for gene expression analysis.

**Results:**

Genetic modification had no effects on the morphology, differentiation ability, or immunophenotype of MSCs. Moreover, MSC-FGF21+GLP1 cells exhibited significantly increased secretion of FGF21 and GLP1. In the T2DM mouse model, the transplantation of MSC-FGF21+GLP1 cells ameliorated the changes in blood glucose and weight, promoted the secretion of insulin, enhanced the recovery of liver structures, and improved the profiles of lipids. Moreover, FGF21 and GLP1 exerted synergistic effects in the regulation of glucolipid metabolism by controlling the expression of insulin, srebp1, and srebp2.

**Conclusion:**

Stem cell treatment based on MSCs modified to overexpress the FGF21 and GLP1 genes is an effective approach for the treatment of T2DM.

**Supplementary information:**

**Supplementary information** accompanies this paper at 10.1186/s13287-021-02205-z.

## Introduction

Diabetes mellitus (DM) is a complex metabolic disease characterized by chronic hyperglycemia, insulin resistance, and islet β-cell dysfunction [1–3]. DM is listed as one of the top ten global diseases that cause human deaths by the World Health Organization (WHO). At present, there are millions of people in the world with diabetes, and the risk of developing diabetes in the future is very high [4]. According to a report published in Lancet Diabetes and Endocrinology, the number of people with T2DM will increase from 406 million in 2018 to 511 million by 2030, and this increase will be the result of the continuous increase in global obesity [5]. The treatment of diabetes is a long-term process. The long-term use of various chemicals has many limitations and leads to adverse effects and even serious complications, such as hypoglycemia and lactic acidosis. To date, there are no drugs to cure diabetes. Chemicals can only control blood glucose. The use of insulin is expensive, and it needs to be injected every day. It is not easy for patients to adhere to this treatment, which requires strict control of the administration time and dose; otherwise, hypoglycemia can easily occur. In addition, chemicals cannot repair damaged tissues. Therefore, there is an urgent need to develop new therapies.

Insufficient secretion of endogenous hormones, cytokines or enzymes, decreased activity, or functional defects are closely related to the occurrence of metabolic diseases. For example, insulin resistance and relative insufficiency of islet cell secretion are the core causes of diabetes [6]. Therefore, the key molecules in the balance of glucose and lipid metabolism are also the key targets of drug development for metabolic syndrome. Among these molecules, glucagon-like polypeptide 1 (GLP1) and FGF21 have become important drugs for the treatment of diabetes and obesity [6–10]. GLP1, which is secreted by the L cells of the ileum and colon, plays an important role in maintaining glucose homeostasis and other physiological processes [10]. GLP1 receptor agonists can promote glucose-dependent insulin secretion to treat T2DM [11]. GLP1 receptor agonists mainly promote insulin release by activating the GLP1 receptor. The GLP1 receptor enhances calcium influx via calcium ion channels and calcium ion release from the endoplasmic reticulum through the cAMP/PKA pathway, activates calmodulin, and finally promotes insulin exocytosis [12]. Studies have shown that GLP1 does not promote insulin secretion when blood glucose levels are lower than 4.5 mmol/L. Therefore, GLP1 receptor agonists can reduce the risk of hypoglycemia and maintain the balance of blood glucose [13]. In addition to their hypoglycemic effects, GLP1R agonists also protect and repair β-cells by inhibiting the secretion of glucagon and stimulating the proliferation and regeneration of beta cells [12, 14]. Fibroblast growth factor 21 (FGF21) is produced by tissues involved in metabolism, such as the liver, adipose tissues, skeletal muscle, and pancreas, and has been suggested to improve metabolic diseases and induce weight loss in humans and mice [15–17]. Recently, a synthetic FGF21 variant, LY2405319, has been shown to reduce low-density lipoprotein (LDL) cholesterol and triglyceride levels, increase adiponectin levels, improve fasting insulin levels, and induce weight loss in obese patients with type 2 diabetes [15]. FGF21 administration is associated with decreased levels of sterol regulatory element-binding protein (SREBP), which is necessary for FGF21-induced thermogenesis [18]. Chronic treatment with recombinant FGF21 reduces serum and hepatic triglyceride levels and improves fatty liver in obese mice by inhibiting the adipogenesis gene SREBP-1 [7]. In addition to Srebp-1, Srebp-2 has also been identified as a target of FGF21, and Srebp-2 acts as the main regulator of cholesterol biosynthesis by preferentially activating the transcription of key cholesterol-producing genes in the liver [19]. All evidence suggests that FGF21 is a promising cytokine for the treatment of metabolic disorders. Interestingly, GLP1 therapy can also activate the iNKT-FGF21 axis in vivo, which contributes to weight loss [20]. That is, GLP1 can regulate the expression of FGF21 or play a synergistic role with FGF21 in regulating glucose and lipid metabolism.

An attractive strategy for treating diabetes is stem cell therapy. Stem cells have the ability to self-renew and can differentiate into various types of cells. In T2DM, injected pluripotent stem cells can differentiate into β-islet cells, thus improving the symptoms of diabetes. At present, the aim of the use of stem cell therapy for treating T2DM is to induce stem cells to differentiate into islet-like cells without considering tissue repair and insulin resistance. Mesenchymal stem cells (MSCs) are derived from different adult tissues and have long-term self-renewal abilities. Under specific conditions, MSCs can differentiate into a variety of cell types [21]. Under different physiological and pathological conditions, MSCs can maintain homeostasis through multidirectional differentiation. MSCs secrete a large number of cytokines and transmit chemical signals between cells, and MSCs are widely used in regenerative medicine research [22–24]. New treatment methods based on MSCs have satisfactory therapeutic effects in clinical applications. In the clinical treatment of diabetes, preliminary animal experiments and clinical evidence have proven that MSC infusion can effectively decrease blood glucose levels and insulin sensitivity in muscle, fat, and liver tissue and reduce complications, such as diabetic nephropathy and diabetic foot and lower extremity vascular disease. More importantly, in the application of MSCs in clinical research, no serious adverse reactions have been reported, which indicates that MSCs are safe for clinical treatment [25–29]. However, there is a problem in the clinical application of MSCs in diabetes. Due to the different sources of MSCs, there may be significant differences in the cell characteristics and therapeutic effects of MSCs. Therefore, new adjuvant therapy is needed to solve the problems mentioned above.

To solve these problems associated with MSCs, we used lentiviral particles to infect adipose-derived mesenchymal stem cells and induce high expression of the GLP1 and FGF21 genes. The therapeutic effect of genetically modified MSCs on diabetes was verified by infusion of diabetic mice. The results showed that FGF21 and GLP1 gene-modified MSCs could significantly improve insulin resistance and promote β-cell function recovery. In addition, gene-modified MSCs possess significant hypoglycemic and hypolipidemic activities, which may be due to the decreased expression of SREBP1 and SREBP2 and the increased expression of insulin in lipid metabolism. Based on these results, we have developed new MSCs for the treatment of metabolic disorders, and these MSCs will have the potential to fundamentally improve diabetes.

## Materials and methods

### Construction of the fgf21-glp1-IgG4fc lentiviral expression plasmid

pGSI-fgf21-glp1-IgG4fc (synthesized by Taihe Biotechnology) was used to subclone the fgf21 and glp1-IgG4fc genes into the lentivector pCDH-EF1 (Addgene) with the EF1α promotor. The amino acid sequence and the nucleotide sequence of the fgf21+ glp1-IgG4fc gene are listed in the supplementary materials. The primers used to amplify the cDNA of the fgf21+ glp1-IgG4fc gene (forward 5′- CGCGGATCCGCCACCATGGACTCGGACGAGACC -3′, reverse 5′- ACGCGTCGACTCATTTACCCGGAGACAG -3′) were synthesized by TsingKe (Beijing). The glp1-igg4fc gene is abbreviated as “glp1 gene”.

### Lentivirus production

Lentiviral vector plasmids and packaging plasmids (psPAX and pMD.2G) were purchased from Addgene. Lentiviral particles carrying pCDH-EF1-FGF21, pCDH-EF1-FGF21+GLP1, and pCDH-EF1-GLP1 were produced through the transfection of HEK293T (ATCC) packaging cells with a 3rd generation plasmid system. HEK293T cells were transfected with 24 μg of plasmids, 48 μl of Lipofectamine LTX, and 24 μl of PLUS reagents, and the proportions of the pMD.2G, psPAX, and pCDH-EF1 plasmids were 1:2:3. The supernatants were collected at 24 and 48 h after transfection, filtered through 0.45-μm filters, and harvested by ultrafiltration with a 100-kDa spin column (Millipore) at 4 °C and 4000 g for 30 min. The lentiviral particles were aliquoted and stored at − 80 °C until use. The transfection efficiency was determined based on EGFP expression using flow cytometry (Beckman), and the viral titers were determined according to the following equation: virus titer (pfu/mL) = cell number in each well × virus dilution factor × 10/volume of added virus fluid (mL).

### Mesenchymal stem cell culture, flow cytometry analysis, and characterization

Adipose tissue-derived mesenchymal stem cells were donated by Xijing Hospital and cultured in the same way as traditional cells. Briefly, to obtain the upper adipose tissue, healthy adult adipose tissue extracted by liposuction was transferred to a 50-mL centrifuge tube, completely washed with PBS, and centrifuged at 1500 rpm for 5 min. Mixed collagenase (0.2%; type I, II, and IV collagenases = 1:1:1) was prepared, and a 1:1 mixture of adipose tissue to collagenase was added to the mixed collagenase digestion solution. The adipose tissue was digested in a 37 °C shaker for 30 min. The digested adipose tissue was immediately added to α-MEM cell culture medium containing 10% FBS (Gibco), that is, complete medium. To precipitate the cells and tissue clumps, the mixture was centrifuged at 1500 rpm for 10 min. The cells were resuspended using complete medium, and the undigested tissue was removed by nylon mesh. The cells were inoculated in a culture flask and incubated at 37 °C in a 5% CO_2_ incubator. Two days later, the nonadherent cells were discarded, and the adherent cells were washed gently with PBS. The cells continued to be cultured in complete medium.

MSCs were harvested from passage 5 and washed three times with PBS. A total of 1 × 10^6^ cells were incubated with 5 μl ECD-conjugated antibodies, 20 μl FITC/PE-conjugated antibodies, or the relevant isotype control antibodies (Beckman Coulter, CD73-PE B68176, CD90-FITC IM1839U, CD105-PE B92442, CD34-PE A07776, CD45-ECD A07784, IgG1 Mouse-FITC IM0639U, IgG1 Mouse-PE IM0670U, IgG1 Mouse-ECD A07797) for 20 min in the dark at room temperature. Then, the cells were washed three times with PBS and examined by flow cytometric analysis (flow cytometer model: Beckman Coulter EPICS XL). In total, more than 95% of the cells expressed CD73, CD90, and CD105, while 2% or less of the cells expressed CD45 and CD34. The released cells were negative for pathogenic microorganisms, HBV, HCV, HIV, cytomegalovirus, syphilis, and ALT, and the endotoxin levels were found to be within 40 IU/L and 0.5 EU/mL. The total cells were counted, and cell viability (≥ 85%) was determined by Trypan blue staining.

### Transduction of MSCs with lentiviral particles and detection of target gene expression

MSCs (< 3 passages) were transduced with concentrated lentivirus at a multiplicity of infection (MOI) of 40 for 6 h in α-MEM containing 8 μg/ml polybrene. To detect the expression patterns of FGF21 and GLP1 in the MSCs, Western blot analyses of the cellular supernatants were performed using anti-FGF21 and human IgG4-Fc monoclonal antibodies. To further measure the secretion of FGF21 and GLP1, the culture medium (CM) of the MSCs and MSCs transduced with pCDH-EF1-FGF21, pCDH-EF1-FGF21+GLP1, pCDH-EF1-GLP1, or pCDH-EF1-vector lentiviral particles was collected after incubation for 48 h. The FGF21 and GLP1 levels secreted into the MSC culture medium were measured by ELISA (Abcam) according to the manufacturer’s protocol. When collecting the culture supernatant for testing, to ensure that the same sample quantity was collected, we inoculated different kinds of cells at a uniform density and then added the same amount of medium. After 48 h of culture, the same amount of centrifuged culture supernatant was analyzed by ELISA and WB. To test the proliferation of the MSCs, each MSC type was seeded in 96-well plates at 5 × 104 cells/well and preconditioned in culture medium. After 48 h of incubation, 20 μl of CCK-8 was added to each well and incubated for 4 h at 37 °C, and the absorbance was measured at 570 nm with a Quant microplate reader. All the samples were analyzed in duplicate, and the samples with coefficient of variation (CV) values > 15% were excluded.

### Adipogenic and osteogenic differentiation

MSCs were cultured in a 24-well plate in complete α-MEM supplemented with adipogenic- and osteogenic-inducing agents (Sigma Aldrich) at an initial cell density of 1 × 10^4^ cells/well. The adipogenic medium was α-MEM containing 10% FBS, 1 mmol/L dexamethasone, 5 mg/mL insulin, and 100 mmol/L indomethacin. The osteogenic medium was α-MEM containing 10% FBS, 0.1 mmol/L dexamethasone, 50 mmol/L ascorbic acid, and 10 mmol/L β-glycerophosphate. The medium was changed every 3 days. After 2–3 weeks, the cells were washed twice with PBS and fixed with 4% paraformaldehyde at room temperature for 30 min. The intracellular lipid droplets were visualized by oil red staining, and calcium deposits were stained with alizarin red S.

### Western blotting

The cells were washed with PBS buffer and subsequently lysed using cell lysis buffer (Tiangen) with a complete protease inhibitor mix (Biotool). Liver tissue was ground and subsequently lysed using lysis buffer (Tiangen) with a complete protease inhibitor mix (Biotool). The lysates and protein markers were run in SDS-PAGE gels (12% or 15%) and transferred onto nitrocellulose membranes (Millipore). The membranes were blocked with 5% milk in Tris-buffered saline plus Tween 20 (TBST) and exposed to rabbit or mouse primary antibodies (1:3000, Abcam or Cell Signaling). The blots were probed with horseradish peroxidase (HRP)-conjugated goat anti-rabbit (or mouse) IgG (H+L) secondary antibodies and visualized using a Pierce ECL Western Blotting Substrate kit (Thermo Scientific) for signal detection.

### Relative quantitative real-time polymerase chain reaction (RT-PCR)

Total RNA was isolated with TRIzol (Sigma) in a manner that was counterbalanced across the experimental groups. cDNA was synthesized from 1 μg of total RNA with the cDNA Synthesis Supermix (BioScript All-in-One cDNA Synthesis; Biotool). Quantitative real-time PCRs were performed using SYBR Premix Ex Taq (Tli RNaseH Plus) (Takara) in a 7500 Real-Time PCR System (Applied Biosystems). For normalization, the threshold cycles (Ct-values) were normalized to β-actin/GAPDH within each sample to obtain the sample-specific ΔCt values (ΔCt 1/4 Ct gene of interest Ct β-actin/GAPDH). The 2^−ΔΔCt^ values were calculated to obtain the fold expression levels. The primers for the quantitative analyses of the FGF21 gene (forward 5′- ATCGCTCCACTTTGACCCTG -3′, reverse 5′- GGGCTTCGGACTGGTAAACA -3′), GLP1-IgG4Fc gene (forward 5′- CCCCAAAACCCAAGGACACT -3′, reverse 5′- GCCATCCACGTACCAGTTGA -3′), srebp1c gene (forward 5′- CACTGTGACCTCGCAGATCC -3′, reverse 5′- ATAGGCAGCTTCTCCGCATC -3′), insulin gene (forward 5′- TCTCTACCTAGTGTGCGGGG -3′, reverse 5′- GCTGGTAGAGGGAGCAGATG -3′), β-actin gene (forward 5′- CCTGGCACCCAGCACAAT -3′, reverse 5′- GGGCCGGACTCGTCATAC -3′), and GAPDH gene (forward 5′- GGAGCGAGATCCCTCCAAAAT -3′, reverse 5′- GGCTGTTGTCATACTTCTCATGG -3′) were synthesized by TsingKe Company (Beijing).

### Animal experiments

In our study, BKS.Cg-Dock7m+/+Leprdb/Nju mice (T2DM mouse model) were used, and the mice were purchased from the Model Animal Research Center of Nanjing University. Thirty-six male BKS mice aged 6–8 weeks (> 20 g body weight) were randomly divided into six groups. Each group contained six mice housed in two cages. The experiment was divided into six groups. The control group was intraperitoneally injected with 100 μl saline. The liraglutide group was injected with 100 μl of liraglutide drug (0.5 mg/kg) twice a week until the end of the experiment. The MSC group (containing pCDH-EF1-vector lentiviral particles), MSC-FGF21 group, MSC-FGF21+GLP1 group, and MSC-GLP1 group were injected with 1× 10^6^ MSCs suspended in 0.1 mL of physiological saline once a week for 3 weeks. Before each injection, the cells were passed through a 70-μm cellular sieve and, then, the cells were injected into the mice at 3–5 time points on each injection day, at intervals of approximately 10 min. The drugs were administered by intravenous injection. The glucose levels in the blood obtained from the tails was measured every week during the experiments. On day 28, peripheral blood was collected from the retro-orbital sinus of each mouse.

### Glucose-stimulated insulin secretion (GSIS)

The rat INS-1 pancreatic β cell line was purchased from CCTCC (China Center for Type Culture Collection). The cells were cultured at 37 °C in a humidified atmosphere containing 5% CO_2_. The culture medium was RPMI 1640 medium containing 11 mM glucose and supplemented with 10% FBS, 10 mM HEPES, 100 U/ml penicillin, 100 μg/ml streptomycin, 2 mM L-glutamine, 1 mM sodium pyruvate, and 50 μM mercaptoethanol. The culture medium was replaced every second day, and the cells were passaged once a week following trypsinization.

To determine the effect of genetically modified MSCs on GSIS, INS-1 cells were seeded onto 12-well plates and cultured for 24 h. Then, the cells were washed two times with Krebs-Ringer bicarbonate buffer (KRBB, 129 mM NaCl, 4.8 mM KCl, 1.2 mM MgSO_4_, 1.2 mM KH_2_PO_4_, 2.5 mM CaCl_2_, 5 mM NaHCO_3_, 0.1% BSA, 10 mM HEPES (pH 7.4), and 2.8 mM glucose) and starved for 2 h in KRBB. The cells were incubated in fresh KRBB containing different MSC-conditioned media for 1 h in the presence of glucose. The supernatants were collected to measure the insulin concentration.

### Fasting glucose and glucose tolerance tests

For the weekly fasting glucose test, the mice were starved overnight to assess glycemia. At the end of the experiment, after overnight fasting, the mice were administered glucose (1 g/kg) by oral gavage, and blood samples were collected from the tail vein to determine the glucose levels. Glycemia was assessed using an Accu-Chek glucometer (Roche, Basel, Switzerland, http://www.roche.com), and the area under the curve was calculated.

### Statistical analysis

All the statistical analyses were conducted using SPSS software. The data were analyzed using one-way ANOVA followed by Tukey’s post hoc test or two-way ANOVA followed by Bonferroni’s post hoc test to determine the differences among the means of the treatment groups. *P* < 0.05 was considered significant.

## Results

### Morphological and immunophenotypic characterization of adipose-derived MSCs

**Fig. 1:**
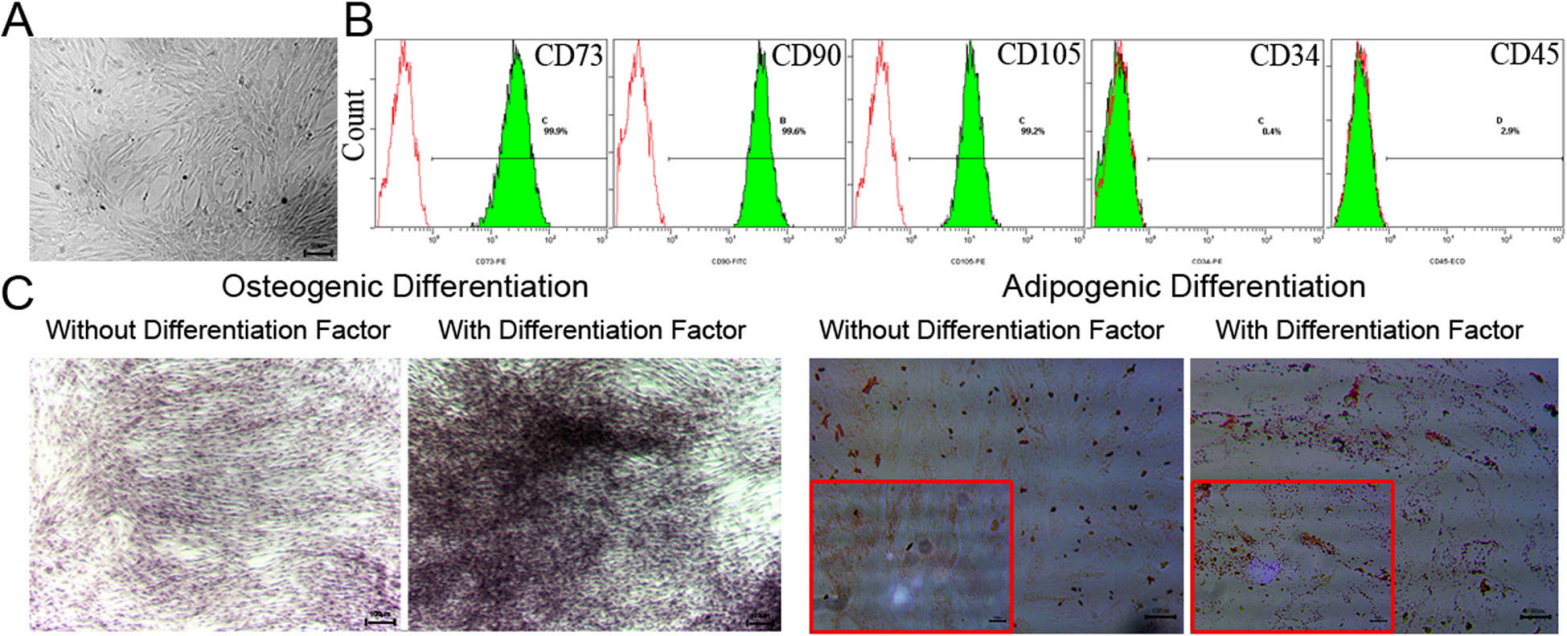
Morphology and multilineage differentiation capacity of MSCs. **a** Adipose-derived MSCs showed a homogeneous spindle-shaped morphology. Bar = 100 μm. **b** Flow cytometric analysis of the phenotypic characterization of MSCs. The phenotypes of CD73, CD90, CD105, CD34, and CD45 expression by MSCs were detected by flow cytometry. The green lines indicate the fluorescence intensity of cells stained with the corresponding antibodies, and the red lines represent isotype-matched negative control cells. **c** Osteogenesis was examined by alizarin red S staining for mineral nodule deposition. Adipogenesis was observed by the presence of lipid vesicles and confirmed by oil red O staining. The picture in the red box shows the enlarged observation of lipid droplets in the cell. Bars = 100 μm

MSCs isolated from human adipose tissue showed a spindle-like morphology similar to that of fibroblasts under phase contrast microscopy (Fig. 1a). In vitro differentiation analysis confirmed that MSCs could differentiate into osteoblasts and adipocytes (Fig. 1c). To further characterize the adipose-derived MSCs, a panel of surface markers was analyzed by flow cytometry. The adipose-derived MSCs were negative for CD34 and CD45 but positive for CD73, CD90, and CD105 (Fig. 1b).

### FGF21 and GLP1 expression in transduced MSCs

**Fig. 2:**
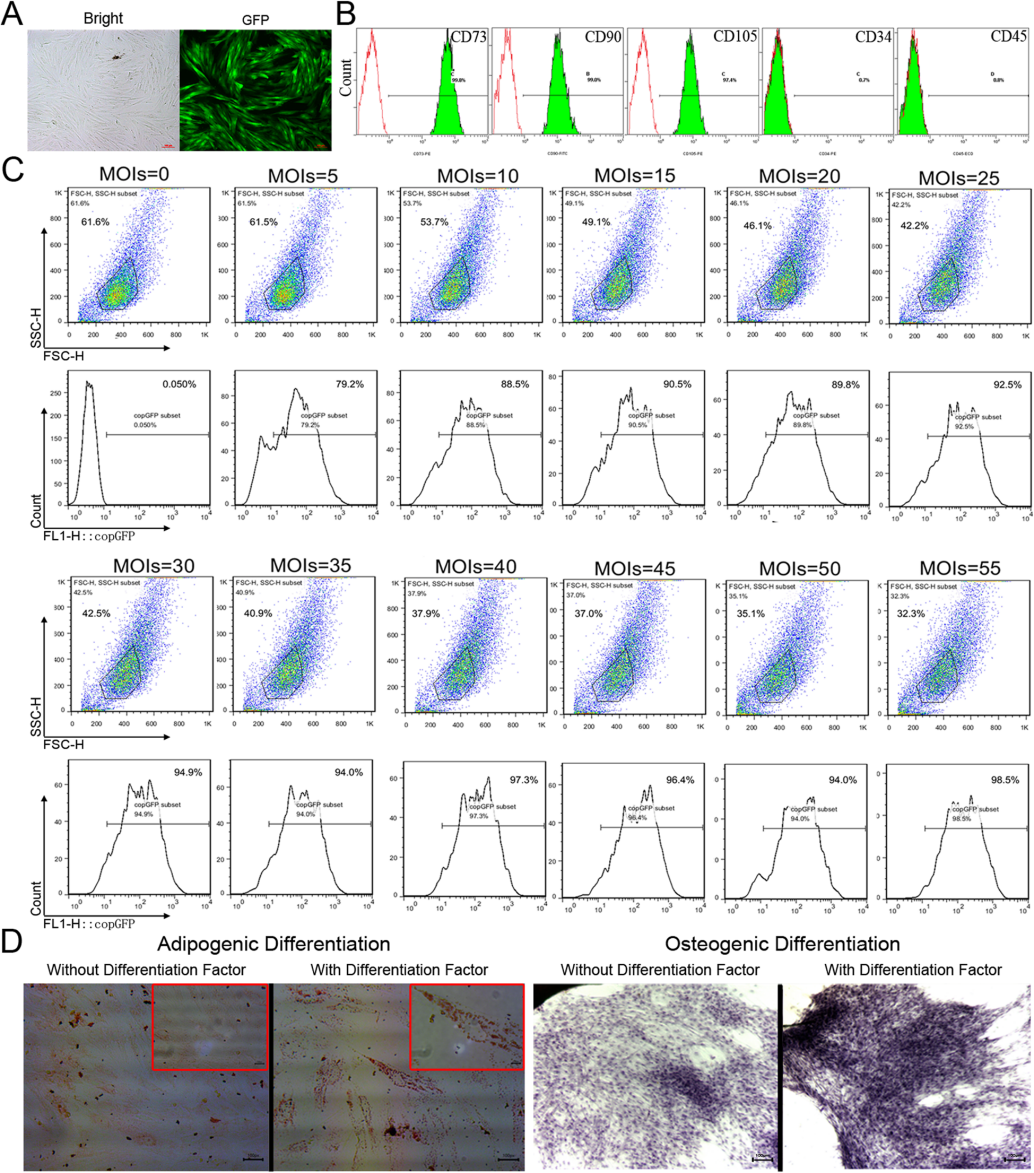
Transduction of MSCs with lentiviral vector particles. **a** Expression of EGFP in MSCs transduced at an MOI of 40 under fluorescence microscopy. Bars = 100 μm. **b** Flow cytometry analysis of phenotype characterization of MSC-FGF21+GLP1. The phenotypes of CD73, CD90, CD105, CD34, and CD45 expression by MSCs were detected by flow cytometry. The green lines indicate the fluorescence intensity of cells stained with the corresponding antibodies, and the red lines represent isotype-matched negative control cells. **c** Analysis of EGFP fluorescence by flow cytometry at 48 h after transduction at different MOIs. The MOI ranges from 0 to 55, at intervals of 5. **d** MSCs transduced with FGF21+GLP1 lentivirus could differentiate into osteoblasts and adipocytes. Osteogenesis was examined by alizarin red S staining for mineral nodule deposition. Adipogenesis was observed by the presence of lipid vesicles and confirmed by oil red O staining. The picture in the red box shows the enlarged observation of lipid droplets in the cell. Bars = 100 μm

To explore the most suitable infection conditions, we infected MSCs with lentivirus particles expressing EGFP (pCDH-EF1-EGFP) and then detected and evaluated these MSCs. EGFP-expressing MSCs were examined by fluorescence microscopy (Fig. 2a). The EGFP expression of the cells was analyzed by flow cytometry 48 h after transduction, and the proportion of EGFP-positive cells ranged from 79 to 98% (Fig. 2c). Flow cytometry analysis showed that when the multiplicity of infection (MOI) was 40, the number of EGFP-positive cells was more than 95%, and the transfection efficiency was not significantly improved between MOIs 40 and 55 (Fig. 2c). Therefore, the optimal MOI of the transduction scheme was 40. Then, the differentiation ability and surface marker expression of MSCs transfected with FGF21 + GLP1 were detected. The results showed that lentiviral particle transduction did not affect the biological characteristics of the MSCs (Fig. 2b, d).

**Fig. 3:**
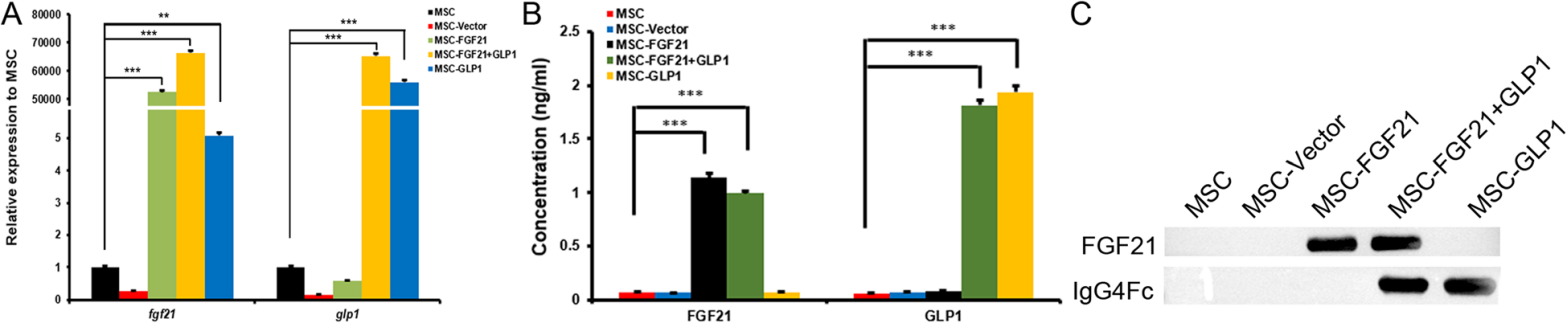
The expression of FGF21 and GLP1 in gene-modified MSCs. **a** Quantitative real-time PCR detected the expression of FGF21 and GLP1 mRNA in FGF21- or/and GLP1-modified cells. Unmodified MSCs and blank vector-modified MSCs were controls. The intracellular β-actin gene was used as a reference gene, **P < 0.01, ***P < 0.001. **b** ELISA was used to analyze the expression of FGF21 and GLP1 in FGF21- and/or GLP1-modified MSC culture medium. Unmodified MSCs and blank vector-modified MSCs were controls, ***P < 0.001. **c** Western blot analysis showed strong FGF21 and GLP1 bands in MSCs transduced with FGF21 or/and GLP1

To further verify the expression of FGF21 and GLP1, we performed quantitative RT-PCR analysis. The results showed that the mRNA expression of FGF21 and GLP1 in the MSCs transfected with FGF21 and/or GLP1 was significantly higher than that in the control MSCs (*P* < 0.05 for all) (Fig. 3a). The contents of FGF21 and GLP1 in the supernatant were detected by ELISA. The results showed that the MSCs in the FGF21 and/or GLP1 gene-modified group could secrete large amounts of FGF21 and GLP1 cytokines (Fig. 3b). In addition, Western blot analysis also showed that the protein expression of FGF21 and GLP1 in the supernatant of the FGF21- or/and GLP1 gene-modified MSCs was significantly increased (Fig. 3c).

### FGF21+GLP1-modified MSC transplantation ameliorated changes in blood glucose and weight in mice with T2DM

**Fig. 4:**
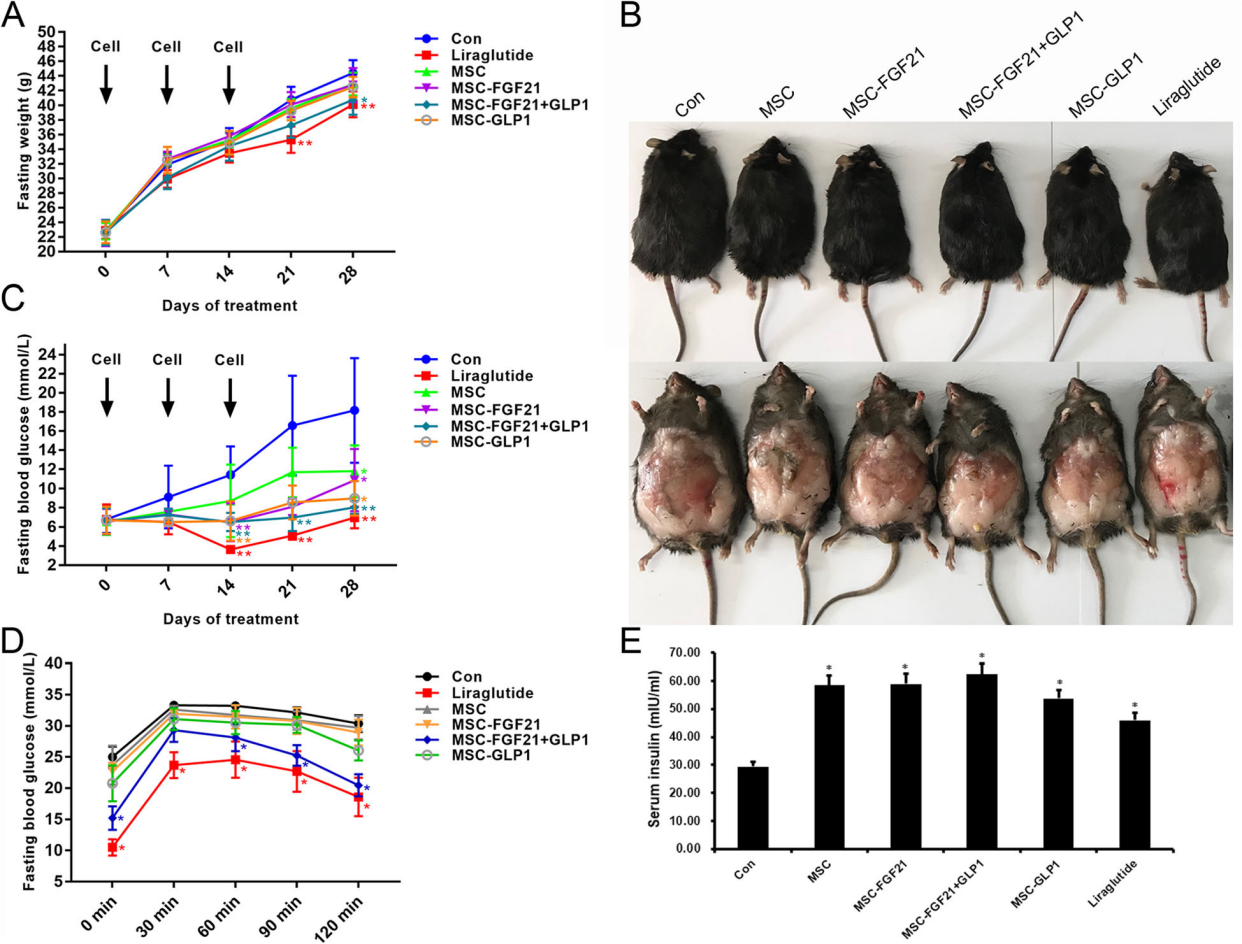
FGF21- and GLP1-modified MSCs reduced blood glucose and weight in T2DM mice. **a** Four-week time course of body weight of BKS mice injected i.p. with saline (Con), MSCs, liraglutide, and FGF21- and/or GLP1-transduced MSCs. The arrow position represents the time of cell injection, *n*=6, *P < 0.05, **P < 0.01, compared to Con. **b** Gross appearance of BKS mice injected i.p. with saline (Con), MSCs, liraglutide, and FGF21- and/or GLP1-transduced MSCs. **c** Time course of the fasting blood glucose concentrations of BKS mice injected i.p. with saline (Con), MSCs, liraglutide, and FGF21- and/orGLP1-transduced MSCs, *P < 0.05, **P < 0.01, compared to Con. **d** Blood glucose concentration from oral glucose tolerance tests in BKS mice injected i.p. with saline (Con), MSCs, liraglutide, and FGF21- and/or GLP1-transduced MSCs; asterisk represents significant differences between groups, *P* < 0.05, compared to Con. **e** The serum insulin levels in each group, *P < 0.05, compared to Con

The effects of MSC-FGF21+GLP1 cells on systemic metabolic disturbances were investigated in our study. BKS.Cg-Dock7m+/+Leprdb/Nju mice (BKS mice), which are deficient in leptin receptor expression, are characterized by obesity, insulin resistance, hyperglycemia, and dyslipidemia. MSCs and a GLP1 analog (liraglutide) were used as the controls. The MSC group, MSC-FGF21 group, MSC-FGF21+GLP1 group, and MSC-GLP1 group were injected with 1 × 10^6^ MSCs suspended in 0.1 mL of physiological saline once a week for 3 weeks. The injected cells were infused into the mice at 3–5 time points on each injection day at intervals of approximately 10 min. After 3 weeks of cell therapy, the trend of weight gain in the MSC-FGF21+GLP1 group was significantly inhibited (Fig. 4a). Compared with that in the untreated mice, the volume of adipose tissue in the BKS mice treated with MSC-FGF21+GLP1 also decreased. Interestingly, MSC-FGF21+GLP1 exerted effects similar to those of liraglutide (Fig. 4b). Moreover, the fasting blood glucose levels of the BKS mice were measured once a week for 4 weeks. As shown in Fig. 4c, MSC-FGF21+GLP1 significantly reduced the fasting blood glucose levels in the BKS mice. The results showed that the hypoglycemic effect of MSC-FGF21+GLP1 was slightly higher than that of other treatments. The same phenomenon was observed in the results of the oral glucose tolerance test (Fig. 4d). In addition, after MSC-FGF21+GLP1 treatment, the plasma insulin level of the BKS mice slightly increased (Fig. 4e). These observations suggested that MSC-FGF21+GLP1 could enhance insulin secretion.

### FGF21+GLP1-modified MSC transplantation could improve lipid disorders in mice with T2DM

**Fig. 5:**
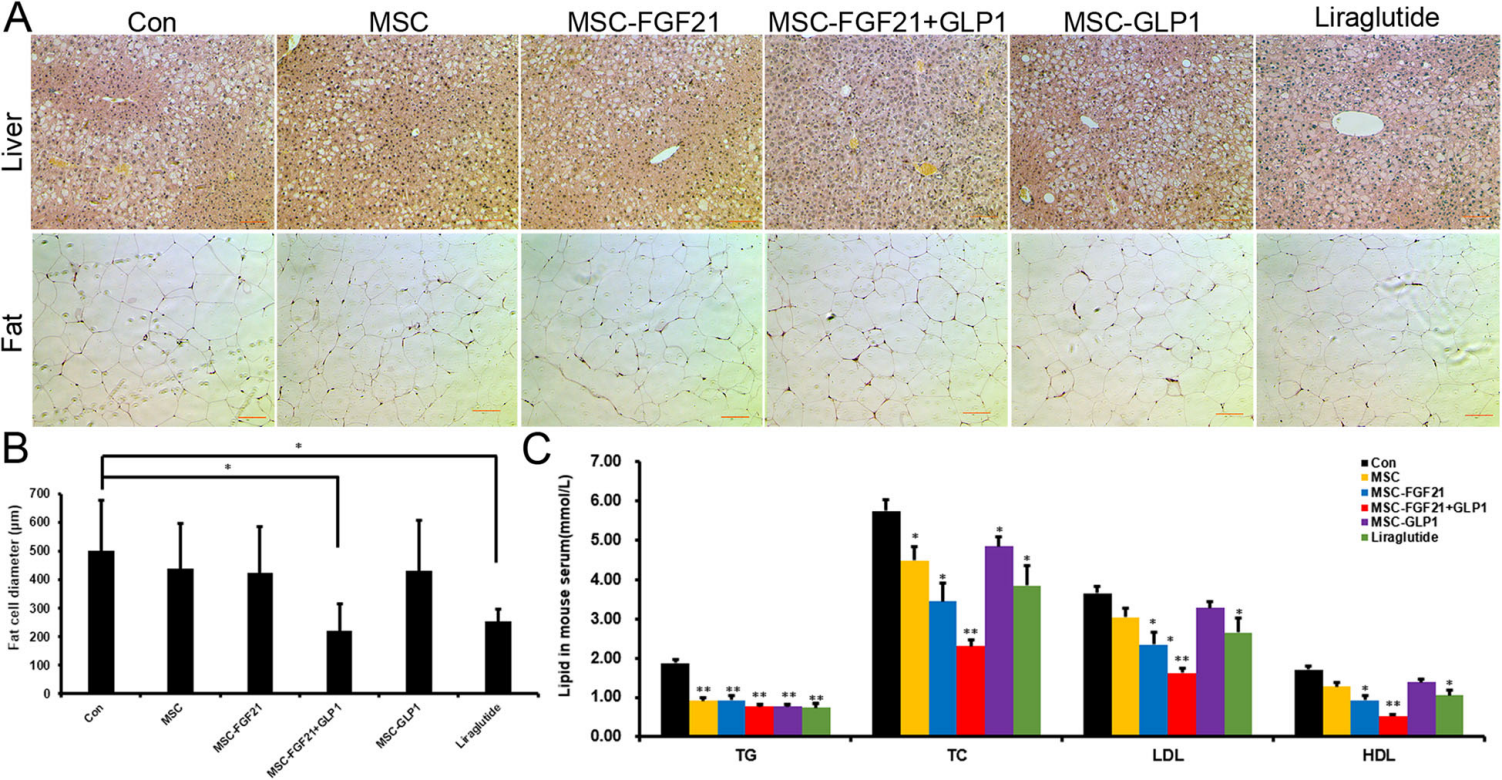
FGF21- and GLP1-modified MSCs could improve lipid metabolism in T2DM mice. **a** Hematoxylin and eosin staining of representative liver and adipose sections obtained from mice from the indicated groups (scale bars = 100 mm). **b** Statistics on the diameter of fat cells, *P < 0.05. **c** The serum TG, TC, HDL-C, and LDL-C levels in the indicated groups, *P < 0.05, **P < 0.01, compared to Con

Histopathological analysis was performed to determine the potential target tissues or organs of MSC-FGF21+GLP1 in T2DM mice. We found that MSC-FGF21+GLP1 could improve the histological structures of the liver and adipose tissue in the BKS mice. The diagnostic index of fatty liver is fatty changes or balloon-like changes in hepatocytes. In pathological sections, there are many blank areas between hepatocytes. The pathological sections from the mice treated with MSC-FGF21+GLP1 showed that the amount of blank area decreased, indicating that the degree of hepatic steatosis was reduced and that the treatment was effective (Fig. 5a). Histological observation of the adipose tissue showed that the size of the abdominal adipocytes in the mice treated with MSC-FGF21+GLP1 substantially decreased, but no significant change was observed in the other mice (Fig. 5a, b). This finding is interesting because the adipose tissue response to MSC-FGF21+GLP1 treatment may partly explain the effects of weight loss and lipid reduction. In accordance with the above results, MSC-FGF21+GLP1 significantly improved the lipid profile of the mice, which was shown by a significant decrease in triglycerides (TGs), cholesterol (TC), low-density lipoprotein cholesterol (LDL), and high-density lipoprotein cholesterol (HDL) (Fig. 5c).

### FGF21+GLP1-modified MSCs significantly suppressed srebp1c transcription and promoted insulin expression

**Fig. 6:**
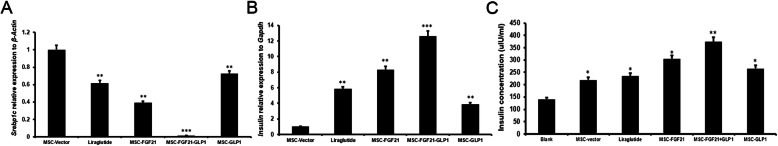
FGF21- and GLP1-modified MSCs could improve glucolipid metabolism in vitro. **a** Quantitative real-time PCR detected the effect of FGF21- and/or GLP1-modified MSCs on the expression of srebp1c mRNA in HepG2 cells. Blank vector-modified MSCs were used as a negative control, and liraglutide was used as a positive control. The β-actin gene was used as the reference gene, **P < 0.01, ***P < 0.001, compared to the MSC vector. **b** Quantitative real-time PCR detected the influence of FGF21- and/or GLP1-modified MSCs on the expression of insulin mRNA in INS-1 cells. Blank vector-modified MSCs were used as a negative control, and liraglutide was used as a positive control. The GAPDH gene was used as the reference gene, **P < 0.01, ***P < 0.001, compared to the MSC vector. **c** Insulin secretion in INS-1 cells incubated in conditioned medium with different modified MSCs. Blank group was KRBH medium without any added reagent. Blank vector-modified MSCs and liraglutide acted as the negative and positive controls, respectively, *P < 0.05, **P < 0.01, compared to the MSC vector

To preliminarily verify the reasons why MSC-FGF21+GLP1 can correct glucose and lipid metabolism, we first detected the expression of SREBP and insulin, the key genes that affect glucose and lipid metabolism. To detect the effects of MSCs on srebp1 gene and insulin gene expression, we used conditioned media from different genetically modified MSCs (FGF21- and/or GLP1-modified) to treat human HepG2 (ATCC) cells and rat INS-1 cells, respectively. As shown in Fig. 6a and b, the supernatant isolated from the MSC-FGF21+GLP1 treatment group significantly inhibited the srebp1c mRNA levels and increased the insulin mRNA levels, and the activity of MSC-FGF21+GLP1 was significantly higher than that of MSC-FGF21, MSC-GLP1, and even liraglutide, the positive control drug. GSIS experiments also confirmed that MSC-FGF21+GLP1 could promote insulin secretion. The results suggested that MSC-FGF21+GLP1 could significantly stimulate insulin secretion by INS-1 cells (Fig. 6c). Therefore, FGF21 and GLP1 double gene-modified MSCs have a significant synergistic effect on regulating glucose and lipid metabolism, especially in regulating srebp1c and insulin gene expression.

### The mechanisms by which FGF21 and GLP1 synergistically improve lipid metabolism

**Fig. 7:**
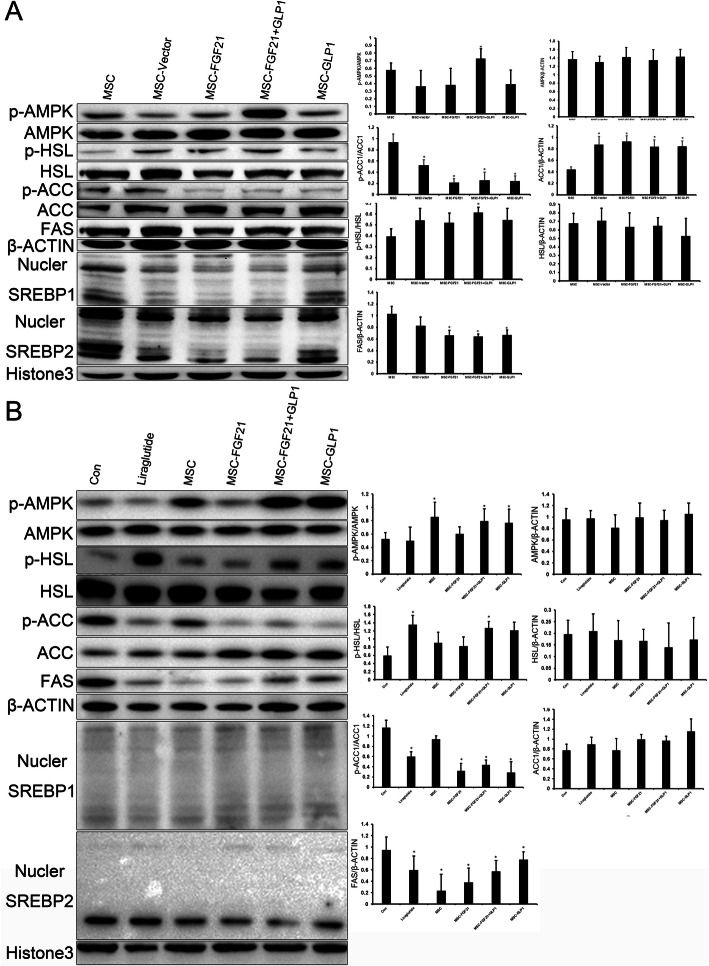
Signaling pathways by which MSC-FGF21+GLP1 regulates lipid metabolism. Western blot analysis verified the signaling pathway by which MSC-FGF21+GLP1 cells regulate lipid metabolism. β-actin was the reference protein for cytoplasmic proteins, and histone 3 was the reference protein for nuclear proteins. p-AMPK, p-ACC, and p-HSL represent the levels of phosphorylation of these proteins. Cytoplasmic proteins were extracted to detect AMPK, ACC, FAS, and HSL. Nuclear proteins were extracted to detect SREBP1 and SREBP2. AMPK, adenosine monophosphate-activated protein kinase; SREBP, sterol regulatory element-binding proteins; ACC, acetyl-coenzyme A carboxylase; FAS, fatty acid synthase; HSL, hormone sensitive lipase. **a** Western blot analysis of the expression of the above genes in HepG2 cells, *P < 0.05, compared to Con. **b** Western blot analysis of the expression of the above genes in the liver, *P < 0.05, compared to Con

To further elucidate the mechanism by which MSC-FGF21+GLP1 synergistically regulates lipid metabolism, we detected the key genes involved in the regulation of lipid metabolism upstream and downstream of the srebp genes. First, we added conditioned medium containing different gene-modified MSCs (FGF21 and/or GLP1) to human HepG2 cells for 48 h. The cytoplasmic proteins and nuclear proteins were extracted, and the protein expression level was detected. Second, because MSCs mainly stay in the liver after being reinfused into mice through the veins, we ground the livers of the mice and then extracted the cytoplasmic proteins and nuclear proteins to detect the expression of the target proteins. As shown in Fig. 7, both in vitro and in vivo experiments showed that MSC-FGF21+GLP1 could significantly increase the level of phosphorylated AMPK, and this effect was much stronger than that of MSCs modified with a single gene. Next, we detected the expression of the SREBP1 and SREBP2 genes in the nucleus. In vitro and in vivo, it was found that the expression of the splicing active protein (base band) and integrity protein (top band) of SREBP1 decreased in the nucleus, while SREBP2 was not affected in the MSC-FGF21+GLP1 group, and the splicing active protein (base band) was significantly decreased in the MSC-FGF21+GLP1 group. In accordance with the above results, the levels of phosphorylated ACC protein and Fas protein downstream of SREBP were decreased, and the level of phosphorylated HSL, an enzyme associated with promoting fat decomposition, was significantly increased. Upon lipolytic stimulation, HSL moves from the cytosol to the surface of lipid droplets where it interacts with perilipin-1 and neutral lipids. Then, the increased number of ATGL-CGI-58 complexes formed following perilipin-1 phosphorylation and docked on small lipid droplets governs PKA-stimulated lipolysis. The association between fatty acid binding protein 4 (FABP4) and HSL represents a further regulatory step. Fatty acid binding to FABP4 and HSL phosphorylation precedes the association of FABP4 and HSL [30].

**Fig. 8:**
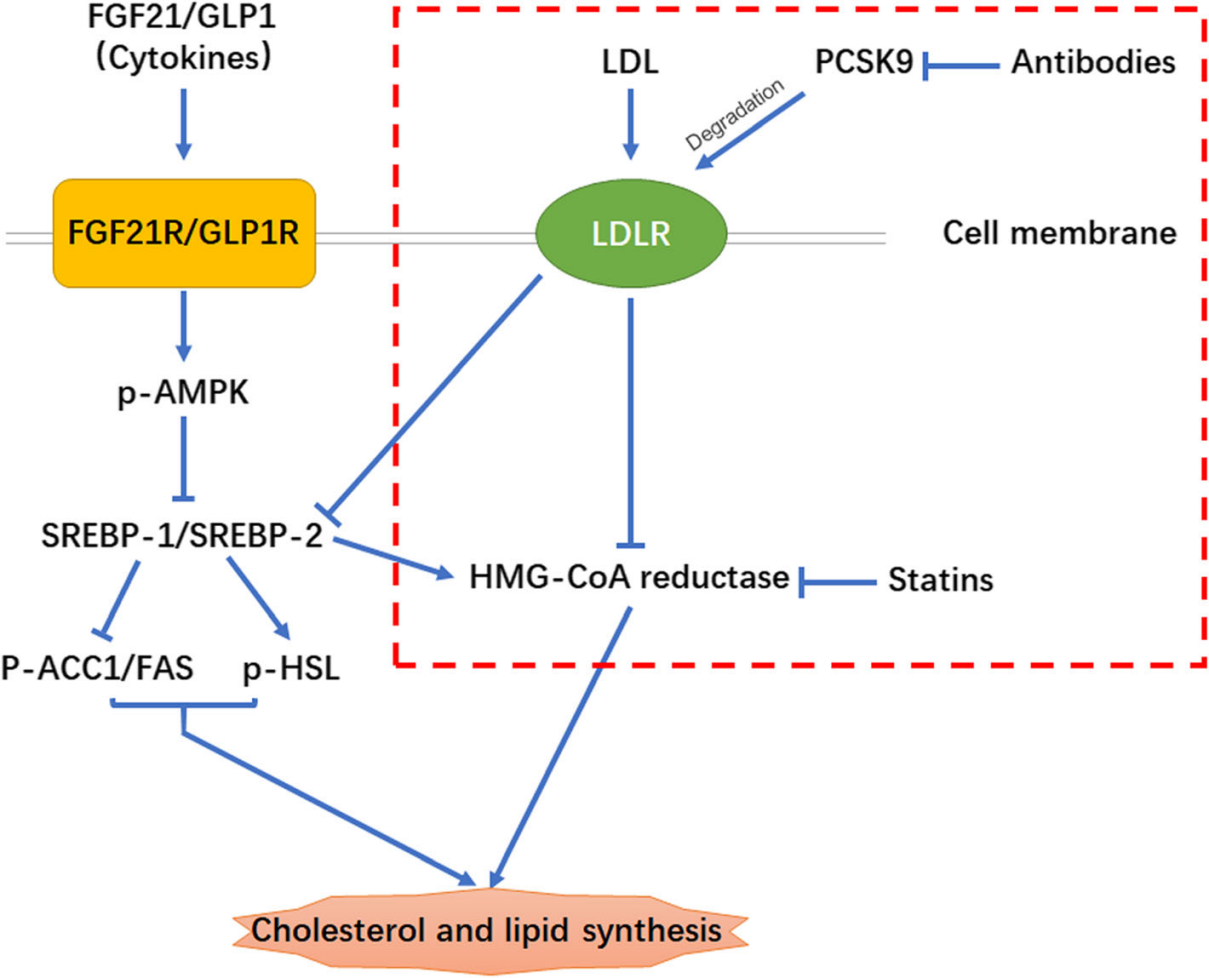
The mechanisms of FGF21 and GLP1 synergistically improve lipid metabolism. The diagram illustrates three existing strategies for regulating lipid metabolism. The red box represents the known mechanisms of metabolic regulation targeting PCSK9 and HMC-CoA reductase. Currently, antibodies and statins are mainly used to block the expression of PCSK9 and HMC-CoA reductase to reduce cholesterol and lipid synthesis. In this study, we focused on cytokines that regulate lipid metabolism. FGF21 and GLP1 bind to their receptors and synergistically enhance the AMPK phosphorylation levels. Activated AMPK further inhibits the expression of the SREBP1/2 genes and mature SREBP1/2 protein in the nucleus. Finally, the expression of enzymes directly involved in lipid metabolism is significantly inhibited

A summary of the mechanism by which FGF21 and GLP1 synergistically improve lipid metabolism is shown in Fig. 8, and the diagram illustrates the three existing methods for the regulation of lipid metabolism. The red box represents the known mechanism of metabolic regulation of PCSK9 and HMC-CoA reductase. At present, antibodies and statins are mainly used to block the expression of PCSK9 and HMC-CoA reductase, thereby reducing cholesterol and lipid synthesis. In this study, we focused on cytokines that regulate lipid metabolism. FGF21 and GLP1 bound to their receptors, and then, they synergistically enhanced AMPK phosphorylation. Activated AMPK further inhibited the expression of the SREBP1/2 gene and mature SREBP1/2 protein in the nucleus and finally regulated the expression of enzymes involved in lipid metabolism.

## Discussion

**Fig. 9:**
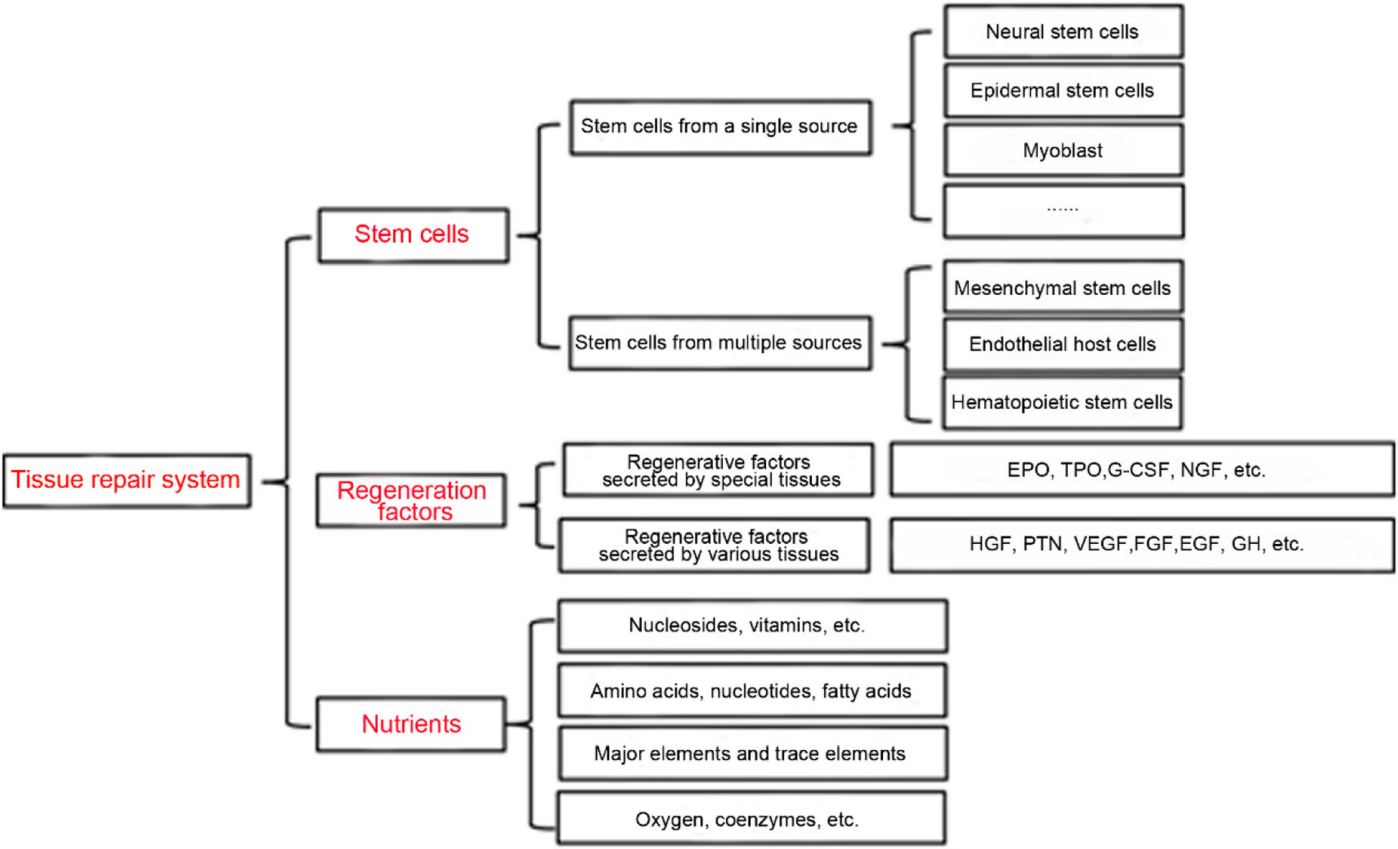
To efficiently change the symptoms of diabetes and achieve tissue repair, stem cells, regeneration factors, nutrients and other comprehensive functions are needed

Diabetes is a chronic disease associated with high morbidity and mortality worldwide. Traditionally, diabetes is divided into type 1 diabetes mellitus (T1DM) and type 2 diabetes mellitus (T2DM); of these, the incidence of T2DM is more than 90% of all cases [31]. Current studies have shown that insulin resistance (IR) and islet β-cell secretion deficiency are the two major pathogenic mechanisms of T2DM [32–34]. Of course, diabetes can also be considered to be a combination of the other three diseases. First, diabetes is an endocrine disease involving disorders of the levels of a variety of hormones, including insulin, glucocorticoids, and adrenal hormones [35]. Second, diabetes is a metabolic disease characterized by abnormal glucose, lipid metabolism, mitochondrial function, nucleic acid regulation, and so on [36–38]. Third, diabetes is a systemic disease characterized by decreased insulin sensitivity in metabolic tissues and organs, which can damage the structure and function of various tissues and organs in the body [39, 40]. Based on the theory described above, to efficiently change the symptoms of diabetes and achieve tissue repair, stem cells, regeneration factors, nutrients, and other comprehensive functions are needed, as shown in Fig. 9. However, the current therapeutic strategies for diabetes mainly focus on the control of glucose and lipid metabolism, and it is difficult to fundamentally improve insulin resistance and tissue repair. To improve this condition, we designed double gene-modified MSCs (FGF21 and GLP1) to achieve multiple repair effects in the treatment of diabetes.

The metabolism of sugar, fat, and protein is the most basic metabolic mechanism in the human body. The mutual regulation of metabolic organs forms a complex regulatory network that involves the neuroendocrine system, growth factors, and enzymes. Generally, there are three types of endogenous molecules involved in the regulation of regeneration factor metabolism. The first type of molecule is hormones, including insulin, glucagon, GLP1, and glucocorticoids. The second group of factors is the cell growth factors with hormone-like functions, including FGF19, FGF21, and FGF23. The third category is involved in metabolic regulation or cell signal transduction enzymes, such as PI3K and HSL. These endogenous hormones, cytokines, or enzymes are closely related to the occurrence of metabolic diseases. In this study, we selected GLP1 and FGF21 as joint repair factors. We chose these two factors mainly because they can interact with each other through the iNKT-FGF21 axis in vivo to regulate body weight [20]. The second reason was that the safety of these two factors in the human body has been demonstrated [9, 15]. There are many drugs approved by the FDA for marketing and drugs in phase II clinical trials, such as liraglutide, dulaglutide, and LY2405319. This ensures the safety of MSC-FGF21+GLP1 in clinical transformation. In this study, we used the GLP1 and FGF21 sequences to refer to dulaglutide and LY2405319, respectively. GLP1 plays a hypoglycemic role by increasing the synthesis and secretion of insulin, inhibiting the emptying of gastric contents, and inhibiting the excitation of the feeding center [10]. In addition, GLP1 can correct the expression of GLUTs in the liver and muscles of patients with T1DM and T2DM, thus changing the glucose intake of cells [41]. FGF21 regulates glucose and lipid metabolism in adipose tissue through endocrine pathways, improves insulin sensitivity and insulin resistance, and stimulates glucose uptake in skeletal muscle through GSIS [7, 8, 41]. FGF21 has been developed as a drug for the treatment of metabolic diseases [6]. FGF21 can improve fatty liver by inhibiting SREBP-1, reducing triglyceride levels in the serum and livers of obese mice, and reducing liver cholesterol production by inhibiting SREBP-2 [18]. In vivo, GLP1 therapy can also activate the iNKT-FGF21 axis, which contributes to weight loss [20]. Therefore, we suggest that GLP1 can further regulate SREBP expression through FGF21 signaling, which may have a synergistic effect on regulating glucose and lipid metabolism. In our study, the combined application of FGF21 + GLP1 significantly reduced the expression of srebp1 and srebp2 and significantly increased the expression of insulin, and these effects were better than those of administration of either alone. The results revealed the synergy between GLP1 and FGF21.

Although GLP1 and FGF21 can effectively alleviate glucose and lipid metabolism in diabetic patients, there are multiple application barriers. The most important obstacle is the extremely short half-life of the drug, and patients need to be injected with large quantities of both drugs daily or weekly, which will result in high cost and drug resistance. Therefore, gene modification of MSCs with FGF21 and GLP1 is a good way to solve these problems. Although the concentrations of FGF21 and GLP1 secreted by MSC-FGF21+GLP1 were low, the effect of MSC-FGF21+GLP1 was better than that of drug therapy alone. MSCs can survive for a long time in vivo and continuously secrete cytokines, which will help patients solve the problem of long-term drug injection. Of course, MSC treatment also has some limitations; its operation is more complex than general drug treatment, the price is generally higher, and these may affect the adherence of the population to this regimen. In addition, due to cell transfusion, there are many uncontrollable factors, so we need to carry out a detailed physical examination before cell therapy. Therefore, we will demonstrate the biosafety and pharmacokinetics of MSC-FGF21+GLP1 in future studies.

The molecular mechanism by which MSCs participate in the treatment of diabetes remains unclear. The possible mechanisms include promoting islet cell regeneration, reducing insulin resistance in peripheral tissues, increasing insulin sensitivity, regulating the immune system, protecting islet beta cells, and improving diabetic complications [26, 42–45]. However, if MSCs are used alone without gene modification, there will be many problems. Diabetic patients are in hyperglycemic states. In the bodies of these patients, high concentrations of blood glucose promote the expression of the PPAR-γ and C/EBP-α genes in transplanted MSCs or autologous MSCs, which makes MSCs more likely to differentiate into adipocytes and osteoblasts. This is not conducive to the repair of damaged islets by MSCs [46, 47]. In addition, the activity of MSCs in patients will decrease as the patients age [48–50]. Therefore, two kinds of gene-modified MSCs (FGF21 and GLP1) were used as a compensatory strategy. Of course, lentiviral transduction might not be approved for clinical trials. We could consider preconditioning MSCs to endogenously express FGF21 or GLP1.

To date, the understanding of the metabolic kinetics of MSCs in vivo mainly comes from animal experiments. Due to the chemotaxis of MSCs to damaged tissues and organs, there are differences in the metabolic kinetics between healthy and diseased subjects. After peripheral intravenous injection, most MSCs remained in the lungs and then reached the liver, kidney, and spleen with blood flow. This may be due to the concentration gradient of substance P that is released from damaged tissues in vivo, since substance P attracts MSCs to migrate to the injured site along its concentration gradient to achieve repair [42]. In this study, MSC-FGF21+GLP1 significantly reduced liver injury, which may be due to this phenomenon. We will discuss the homing sites and survival times of MSC-FGF21+GLP1 in the following experiments.

## Conclusions

In conclusion, this study has shown a new approach that combines FGF21 and GLP1 gene therapy with MSC cell therapy to treat type 2 diabetic mice. We found that infusion of FGF21- and GLP1-modified MSCs could significantly improve insulin sensitivity and glucose metabolism, promote the recovery of liver structure, increase plasma insulin content, and play a synergistic role in regulating glucose and lipid metabolism.

## Supplementary Information

**Additional file 1.** A: The amino acid sequence of FGF21+GLP1. B: The nucleotide sequence of FGF21+GLP1. C: The plasmid profile of pCDH-EF1-FGF21+GLP1 lentiviral vector.

## Data Availability

All the data generated or analyzed during this study are included in this published article.

## Abbreviations

MSC: Mesenchymal stem cell
CM: Culture medium
DM: Diabetes mellitus
T1DM: Type 1 diabetes mellitus
T2DM: Type 2 diabetes mellitus
GLP1: Glucagon-like polypeptide 1
FGF21: Fibroblast growth factor 21
SREBP: Sterol regulatory element-binding protein
GSIS: Glucose-stimulated insulin secretion
TG: Triglyceride
TC: Cholesterol
LDL: Low-density lipoprotein cholesterol
HDL: High-density lipoprotein cholesterol
