# Supplementary figures and images for: Mesenchymal stem cells modified by FGF21 and GLP1 ameliorate lipid metabolism while reducing blood glucose in type 2 diabetic mice
Source: Stem Cell Res Ther. 2021 Feb 15;12:133. doi: 10.1186/s13287-021-02205-z (PMC7885588; doi:10.1186/s13287-021-02205-z)

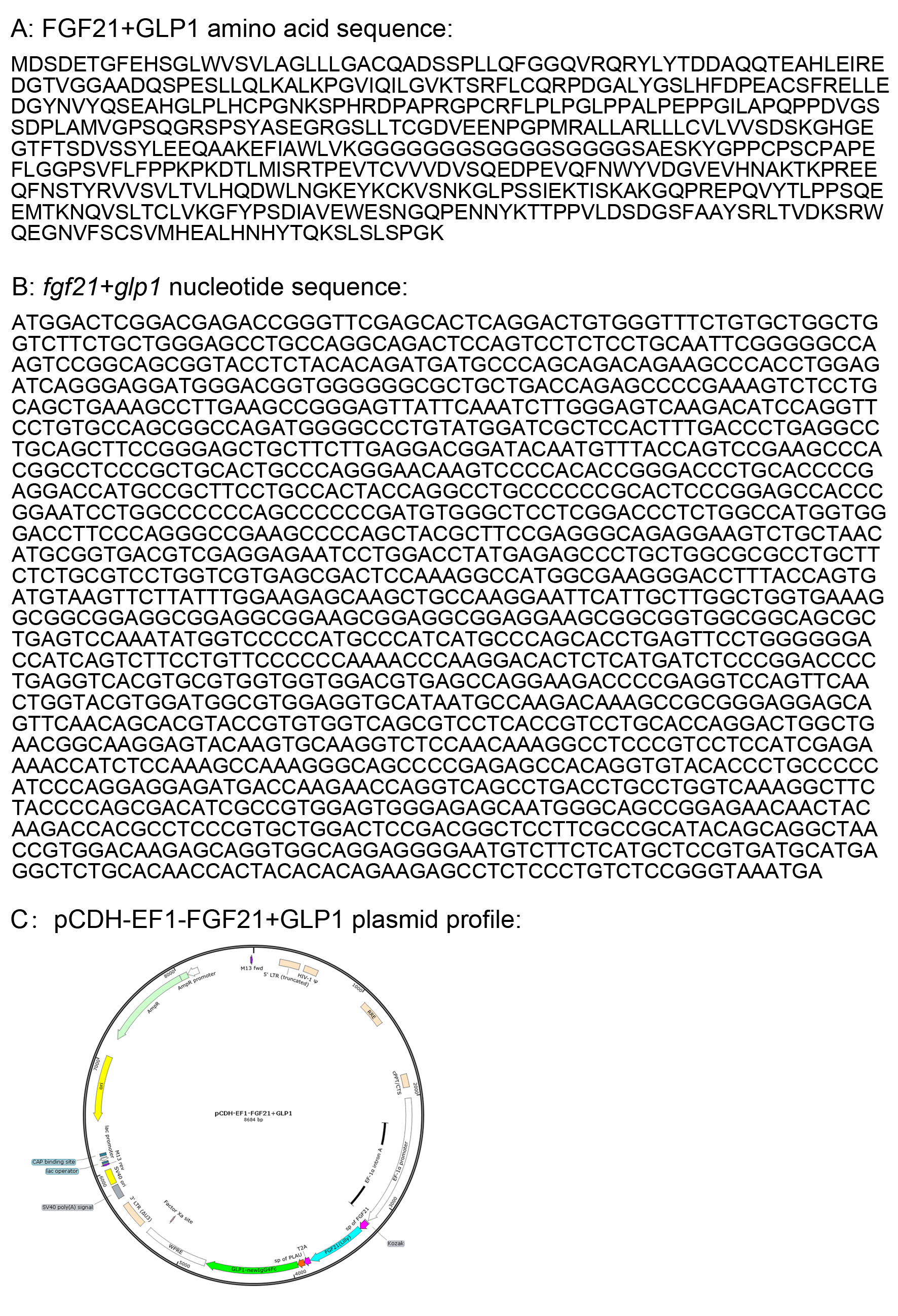

Supplement: Supplementary file 1 — Additional file 1. A: The amino acid sequence of FGF21+GLP1. B: The nucleotide sequence of FGF21+GLP1. C: The plasmid profile of pCDH-EF1-FGF21+GLP1 lentiviral vector. [file 13287_2021_2205_MOESM1_ESM.tif]
